# Data-Based Statistical Analysis of Laboratory Experiments on Concrete Frost Damage and Its Implications on Service Life Prediction

**DOI:** 10.3390/ma15186282

**Published:** 2022-09-09

**Authors:** Fuyuan Gong, Dian Zhi, Jianguo Jia, Zhao Wang, Yingjie Ning, Bo Zhang, Tamon Ueda

**Affiliations:** 1 College of Civil Engineering and Architecture, Zhejiang University, Hangzhou 310058, China; 2 State Key Laboratory for Geomechanics and Deep Underground Engineering, China University of Mining & Technology, Xuzhou 221116, China; 3 Huadong Engineering Corporation Limited, Hangzhou 311122, China; 4 Guangdong Provincial Key Laboratory of Durability for Marine Civil Engineering, College of Civil and Transportation Engineering, Shenzhen University, Shenzhen 518060, China; 5 Institute of Urban Innovation, Yokohama National University, Yokohama 240-8501, Japan; 6 Zhejiang Communications Construction Group Co., Ltd., Hangzhou 311255, China; 7 Ocean Research Center of Zhoushan, Zhejiang University, Zhoushan 316021, China

**Keywords:** concrete, freeze–thaw cycles, laboratory experiment, statistical analysis, durability factor

## Abstract

To meet the requirements of durability design for concrete suffering frost damage, several test standards have been launched. Among the various damage indexes such as deteriorated compressive strength, relative dynamic elastic modulus (RDEM), residual deformation, etc., the concept of a “Durability Factor” (DF) is proposed by many standards to define the frost resistivity of concrete against frost action based on the experimental results from standard tests. Through a review of the literature, a clear tendency of strength/RDEM decay and residual deformation increase is captured with increasing cycles of freezing and thawing. However, tests following different standards finally derive huge scattering quantitative responses of frost resistance. Based on the large database of available laboratory experiments, this study presents a statistical analysis to propose a predictable model to calculate the DF with respect to other material factors. The statistical model is believed to be more convenient for engineering applications since the time-consuming experiment is no longer needed, and it is more precise compared with that developed according to only single experimental results to cover the uncertainties and unavoidable errors in specific tests. Moreover, the formula to calculate the DF is revised into a more general form so as to be applicable for all the laboratory experiments even for those cases without fully following the standards to derive a DF value.

## 1. Introduction

Frost damage is a key durability issue for concrete structures serving in cold and wet regions. Therefore, the frost resistance of concrete shall be evaluated for the purpose of design and maintenance.

From the scientific viewpoint, the mechanisms of concrete frost damage have been widely investigated in the past years [1,2,3,4,5,6,7]. From the physical and thermodynamic point of view, frost damage is caused by several internal pressures that are generated when the pore water freezes, such as the hydraulic [2,8], crystallization, and cryosuction pressure [8,9]. Such pore pressures can be used to quantify the plastic tensile strain (micro-cracks) during freezing expansion based on poro-mechanics [8,10,11,12,13]. The magnitude of plastic tensile strain depends on the local temperature, degree of saturation, and many material variables, such as pore size distribution, porosity, entrained air content, poro-elasticity of the skeleton, tensile strength, etc. [14,15,16]. In addition, this plastic tensile strain accumulates during repeated freezing–thawing actions [17,18]; accordingly, the frost damage will be severe enough to cause obvious degradation in material properties (strength, stiffness, permeability, etc.) [19,20]. It is also widely known that the additional water supply from the ambient environment plays an important role in this damage accumulation [21,22]. Therefore, it indicates that there are many environmental and material parameters that significantly affect the frost damage process, which causes the quantification of frost damage to become rather complicated. These theoretical studies contribute to explaining the mechanism of frost damage in different aspects, but a quantitative evaluation is still lacking. The authors have attempted to develop a comprehensive model for the effective pressure induced by ice formation, and a mesoscale simulation is also conducted by integrating the model with a Rigid Body Spring Model [18]. The simulation depends on the accuracy of the theoretical modeling, and it is still far from engineering application since the code is not open-sourced. As a more typical and applicable way to evaluate frost damage, laboratory experimentation is still a popular methodology.

From the engineering viewpoint, several standard testing methods have been launched in different countries and regions to determine the frost resistance of concrete experimentally, such as ASTM C666 [23], GB/T 50083-2009 [24], JIS A1148:2010 [25], the RILEM CIF test [26], and so on. By conducting laboratory experiments as suggested by these standards, certain characteristics of concrete are measured before and after suffering a treatment of freeze–thaw cycles (FTC), e.g., mass loss, compressive strength, relative dynamic elastic modulus (RDEM), length change, etc. Based on the measured results, some standards bring up the concept of the “Durability Factor” (*DF*) to express the frost resistivity with a calculation formula [23,25,27,28]. It should be noted that the *DF* is just a state parameter describing the situation when the specific criterion is achieved, thus each sample just has one *DF* value. If the experiments stop before the criterion is met, the *DF* can no longer be obtained. Therefore, the engineering way still has some limitations to predict the frost damage process. Besides, the *DF* is considered as an intrinsic material property that is determined experimentally. Therefore, it shall also be able to derive the value of the *DF* from other material properties, such as the water-to-cement ratio (*w*/*c*), compressive strength, etc., which is still lacking, unfortunately.

Considering both the scientific mechanisms and engineering applications of frost damage on concrete, some researchers have tried to propose the integrated scientific and practical ways for damage evaluation. For example, some of the literature discusses the relationships among different damage indexes (strength, stiffness, ultrasonic wave velocity) regardless of frost exposure history, and more stable correlations can be found [17,27,28,29]. These correlations are believed to reflect the mechanical nature of freezing-induced micro-cracks, but the reason is still unclear. Some other researchers tried to explain these damage indexes by using a more fundamental parameter, “plastic tensile strain”, since it is directly caused by freezing expansion, and some mesoscale analyses have also been conducted to prove this idea [17,30]. In addition, using the plastic tensile strain as a linkage, the magnitude of micro-cracking can also be well explained fundamentally through the physical and thermodynamic events [10,31].

As many experimental works following the above-mentioned standards have been conducted to investigate the deteriorated properties of concrete suffering frost damage, these results will be useful to establish the prediction model of *DF* according to other material parameters. Nevertheless, two facts shall be emphasized: (1) the experimental conditions vary in terms of the test standard, and differences even exist when the same standard is referred to with respect to material components, specimen dimensions, FTC scheme, etc. Therefore, a statistical analysis is necessary to ensure the prediction model can be supported by most data; (2) the definition of *DF* requires specific experimental procedure (up to 60% loss of RDEM or 300 FTC) and measurement of a certain damage index (RDEM), while many available experiments cannot satisfy due to the variances as mentioned above. Thus, it is impossible to calculate the *DF* value directly from some data.

In this study, the available laboratory experiments are reviewed in terms of test standards, detailed test variables, as well as damage indexes. The different testing specifications by the standards are summarized where the main differences are the substance and supply method of the cooling medium. Afterwards, the laboratory experimental works are reviewed and the correlations between damage indexes are evaluated. Based on the big data, statistical analysis with both mathematical regression and artificial intelligence is conducted to develop the empirical calculation models of damage index—RDEM—based on the material properties of concrete. Both linear and non-linear models are proposed which can cover over 50% of all the experimental data. Finally, by modifying the expression of the *DF* into a more general form, the intrinsic properties of concrete frost resistivity, the *DF* can be determined by material inputs.

## 2. Review of Laboratory Experimental Data

### 2.1. Test Methods

**Figure 1:**
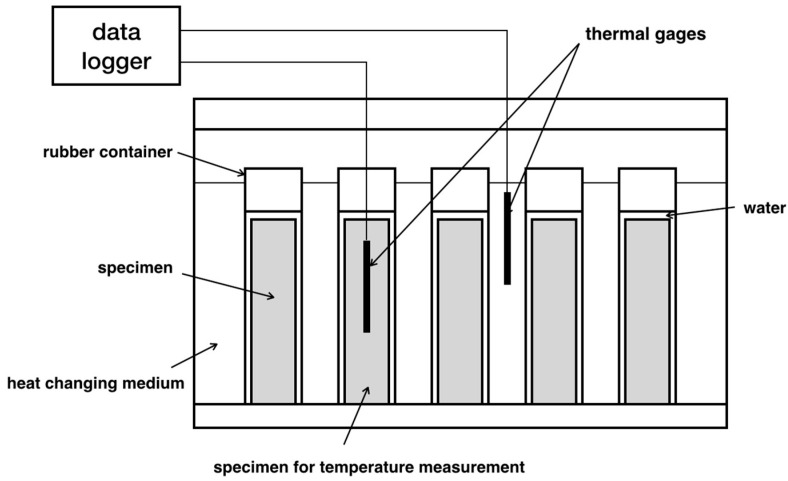
Rapid freezing–thawing testing apparatus according to ASTM C666 [1].

Along with the need to test the frost resistance of concrete becoming critical, various test standards have been launched in the world widely: ASTM C666-03 is recommended by the American Society of Testing Materials (ASTM) as the standard test method for the resistance of concrete subjected to rapid freezing and thawing cycles. It includes two procedures depending on the exposure medium: rapid freezing and thawing in water (A method, see Figure 1) and rapid freezing in air and thawing in water (B method) [23]. A new parameter, the durability factor (*DF*), has been adopted to assess frost damage. Actually, the *DF* is closely related to loss of RDEM, which reflects the level of internal damage.

In China and Japan, GB/T 50083-2009 [24] and JIS A1148:2010 [25] generally follow ASTM C666-03 except for several detailed regulations including specimen preparation and the size of the specimen. Regarding the evaluation of damage, JIS also adopts the DF, while GB introduces a new parameter, *F*, which corresponds to the number of cycles at 5% mass loss or 40% RDEM loss.

Internationally, a relatively complex standard, the CIF test of RILEM TC 176-IDC [26], is generally used, with a longer time of each FT cycle and a three-stage procedure of sample preparation. After subjecting to FTC with one-side water penetration for a certain number of cycles, specimens are evaluated in terms of scaling, moisture uptake, and internal damage. Damage criterion: the concrete is RILEM and adopts a stricter definition of damage when the relative change of the transit time of the ultrasonic wave *R_u,n_* transgresses below 80%.

In contrast, the International Federation for Structural Concrete (fib) does not provide a detailed method for testing but offers an overall description of the damage mechanisms instead [32]. Table 1 lists several main characteristics of the testing methods mentioned above. Several conclusions can be drawn: (1) there are three primary nondestructive examination variables, namely RDEM, mass loss, and length change; (2) as an indicator of internal structure, RDEM is the most recommended parameter to estimate frost damage in general, which is calculated by the transverse frequency (RDEM = (*F_n_*/*F*_0_)2, where F is the transverse frequency); (3) similar criteria are adopted to determine when to terminate the freezing–thawing test, such as 300 cycles or a certain damage level; (4) the main difference of each standard is the cooling medium and its supply method (water or air, all surfaces, or one surface). However, since each standard requires different testing conditions and procedures, it is rather difficult to compare the frost resistance of concrete materials using different testing methods.

### 2.2. Test Variables

Following different test standards as described earlier, there are many laboratory tests available. These experimental data are collected, reviewed, and summarized. All the experimental conditions and material properties are listed in Table 2. The test parameters vary a lot with respect to both material and experimental conditions, such as the number of FTCs, concrete mix proportions (e.g., water-to-cement ratio, air ratio, and compressive strength), and the minimum temperature of each cycle. As summarized in Table 2, experiments by different researchers are generally based on various testing methods. However, even those tests following the same test standard are still flexible in terms of material and experimental conditions. This fact leads to considerable difficulties in comparing the resistance of concrete to FTCs directly by damage criteria. For example, the minimum temperature of each FT cycle was set with a large range (from −35 to −15 °C) even when the same GBJ82-85 standard was adopted.

### 2.3. Test Results

#### 2.3.1. Indexes Change with FTC Number

**Figure 2:**
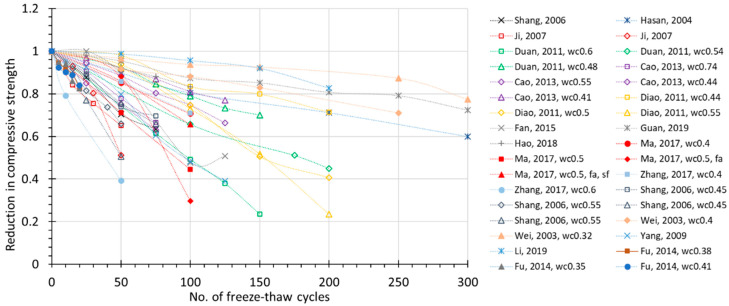
Relative reduction in compressive strength during FTC for non-air-entrained concrete (for the same literature, *wc*—water to cement ratio; *fa*—fly ash added; *sf*—silica fume added).

**Figure 3:**
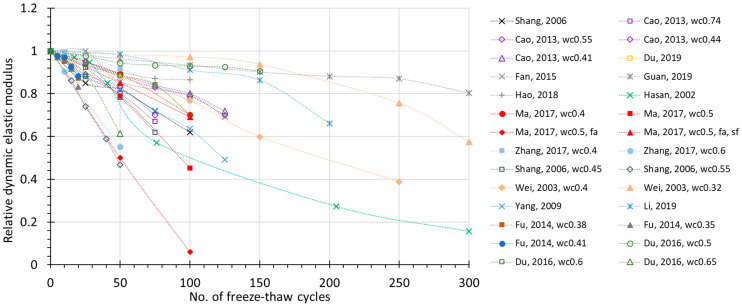
Relative dynamic elastic modulus (RDEM) during FTC for non-air-entrained concrete (for the same literature, *wc*—water to cement ratio; *fa*—ly ash added; *sf*—silica fume added).

**Figure 4:**
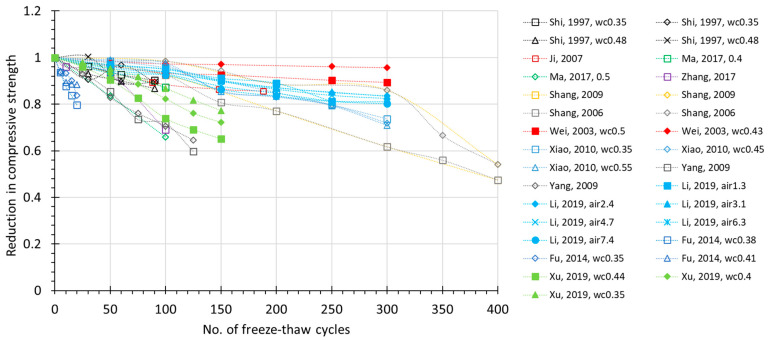
Relative reduction in compressive strength during FTC for air-entrained concrete (for the same literature, *wc*—water to cement ratio; *air*—air content).

**Figure 5:**
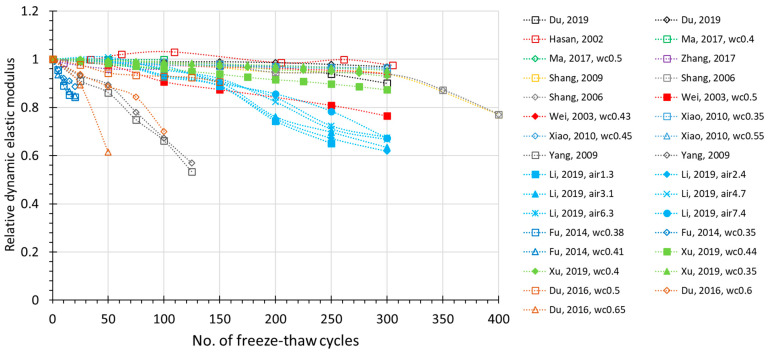
Relative dynamic elastic modulus (RDEM) during FTC for air-entrained concrete (for the same literature, *wc*—water to cement ratio; *air*—air content).

The FTC number-dependent change of several prospective damage indexes are plotted in Figure 2, Figure 3, Figure 4 and Figure 5, where all the available experimental results are demonstrated, including non-air and air-entrained concrete. Compressive strength (*f_c_*) and relative dynamic elastic modulus (RDEM) are chosen as the basic indicators of damage for the reason that they have been widely used in established studies. Moreover, these two indexes both reflect internal frost damage, so it is convenient to analyze the data of *f_c_* and RDEM synchronically.

From the figures, it can be observed that as the number of freezing–thawing cycles increases, the compressive strength and relative dynamic elastic modulus both show a clear degradation trend. However, the scattering data (Figure 2) shows that compressive strength may decrease to 40% in only 50 cycles, or it may decrease to 80% after 300 cycles, which is due to the water-to-cement ratio. Similarly for RDEM in Figure 3, it may drop to less than 10% in only 100 cycles, but it can also remain at 80% even after 300 cycles. This is also true for the air-entrained case in Figure 4 and Figure 5. Most of the test data in Figure 2 and Figure 3 show a linear relationship between the indexes and the number of freezing–thawing cycles, and it can be observed that the higher water-to-cement ratio may lead to a faster speed of deterioration, which will be discussed in detail in Section 3.2. It should be noted that the number of freezing–thawing cycles varies significantly from test to test, and not all experiments were strictly conducted under the guideline of testing standards to calculate the *DF* value. As a result, only about 60% of them reached the required number of freezing–thawing cycles (details are given in Appendix A). As for the air-entrained concrete, the quality of entrained air voids fluctuates due to the different types and materials of the air-entraining agent adopted and the uncertainty involved during the process. As shown in Figure 4 and Figure 5, some air-entrained concretes suffer a slower degradation of indexes, whereas others are still easily damaged, suggesting that in some experiments, the entrained air voids fail to meet the requirement, and those unqualified data will be removed for further discussion.

**Figure 6:**
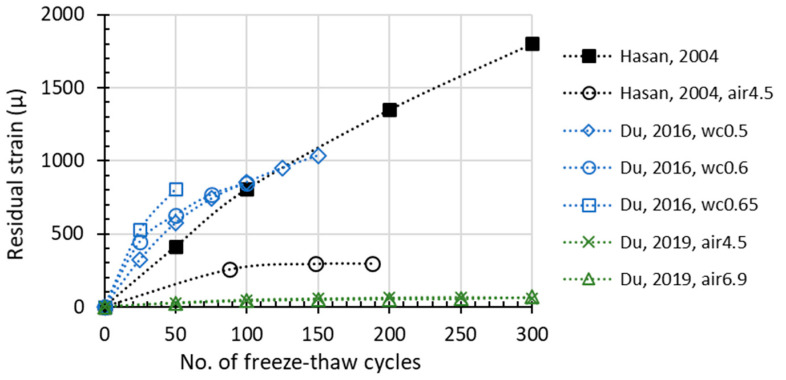
Residual strain (length change) development during FTC (“air” in the legend means entrained air amount, others are non-air-entrained concrete).

Compared with *f_c_* and RDEM, which are the most widely adopted parameters of frost-damaged concrete, residual strain is rarely used in past research. However, the degradation of concrete material is attributed to the initiation and development of meso-cracks, which will lead to unrecoverable plastic tensile deformation and finally result in the measurable residual strain or length change of the whole specimen. In terms of such physical mechanism, residual strain is also considered an important indicator of damage. ASTM specifies the length expansion as one of the several essential measurements after FTCs, and length change is recommended as a damage criterion accordingly. The available test results pertaining to residual strain are illustrated in Figure 6. Since the database of residual strain is not as much as for compressive strength or RDEM, it is rather difficult to conclude a certain degradation mode for plastic strain at this stage. Based on the limited data, the residual strain propagation with the FTC number shows a rather non-linear behavior. Moreover, the effect of an air-entraining agent (AEA) on frost resistance shows some scattering. For instance, the AEA may reduce the residual strain up to 300 micro or less than 50 micro according to Hasan et al. and Du et al. [17,49]. In Figure 6, by introducing AEA, Du’s data [17,49] show more effective improvement in the frost resistance while Hasan’s results [17,49] show less impact, which again proves the fluctuating quality of AEA.

#### 2.3.2. Correlations among Damaged Properties

**Figure 7:**
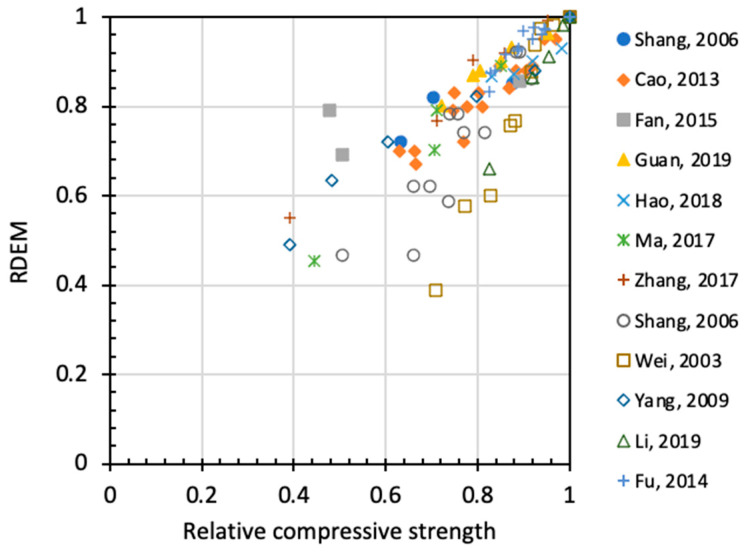
Relation between RDEM and relative compressive strength for non-air entrained concrete.

**Figure 8:**
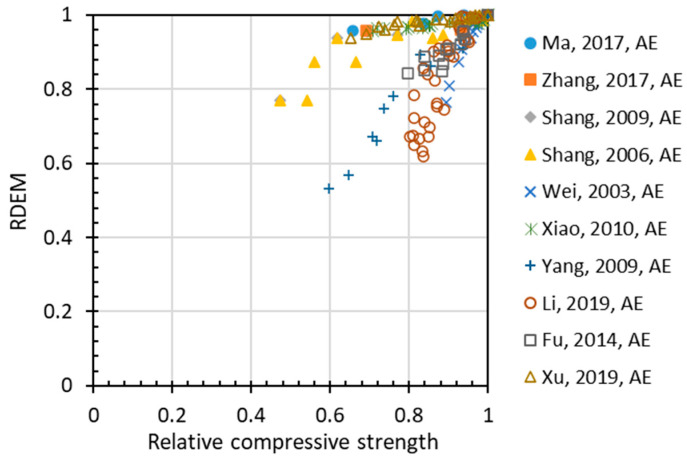
Relation between RDEM and relative compressive strength for air-entrained concrete.

The above-mentioned damage indexes have been proposed for describing the frost damage of concrete, and there have been numerous experiments on the behavior of concrete under freezing–thawing cycles. However, relationships between different indexes have never been discussed systematically. Among these relationships, the most important one is the correlation between relative compressive strength (*Rf_c_*) and relative dynamic elastic modulus. Figure 7 plots the relationship between RDEM and the relative compressive strength of non-air-entrained concrete, in which a strong correlation is found except for some deviant cases [42,43]. For air-entrained concrete, as shown in Figure 8, the relationship between the two indexes has stronger patterns due to the presence of air voids of various qualities.

**Figure 9:**
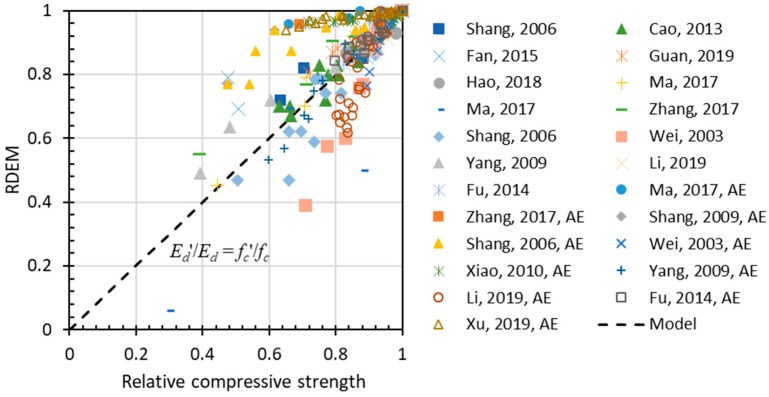
Simple correlation between RDEM and relative compressive strength for both air and non-air entrained concrete.

In Figure 9, simple correlation is drawn for both air and non-air-entrained concrete. It yields that most experiment results correlate with the linear relationship between relative compressive strength and relative RDEM, and the slope can be regarded as one in general. In fact, both compressive strength and RDEM tests are sensitive to various factors during experiments, including laboratory conditions, operation procedure, and so on. For the sake of convenience, the authors adopted a linear model here.

**Figure 10:**
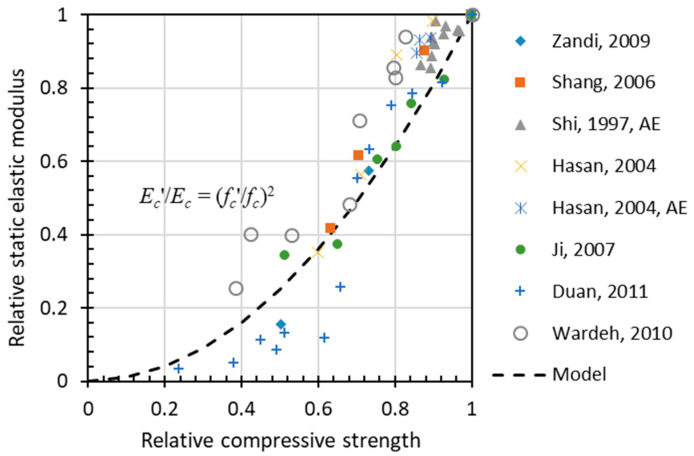
Simple correlation between relative Ec and relative fc.

Other than the dynamic modulus, Figure 10 shows a regressed quadric line of the correlation between the relative static elastic modulus (*E_c_*) and the relative compressive strength (*f_c_*). Comparing Figure 9 and Figure 10, the static elastic modulus has a faster deterioration compared with that for dynamic elastic modulus.

**Figure 11:**
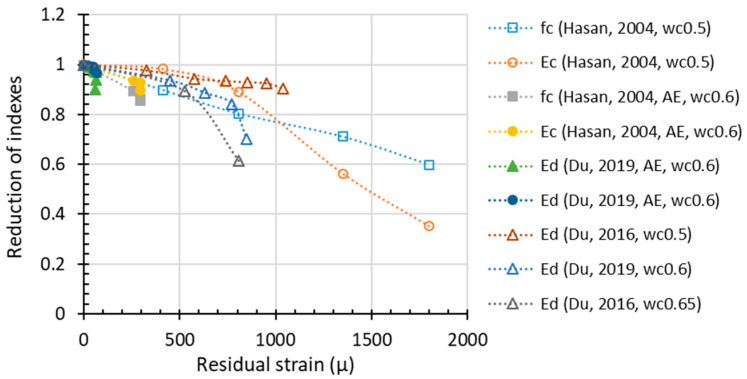
The reduction in mechanical properties in terms of residual strain.

The unrecoverable plastic tensile deformation in the material caused by freezing–thawing cycles can be measured as a residual strain in experiments. Unlike compressive strength and dynamic/static elastic modulus, residual strain is a local measurement, which reflects the micro changes in a relatively small region. In other words, residue strain is the closest to the mechanisms among the indexes of the material deterioration during freezing–thawing cycles, and it exactly explains the degradation of other material properties, including compressive strength, static, and dynamic elastic modulus. As shown in Figure 11, compressive strength, static elastic modulus, and dynamic elastic modulus reduce gradually with the increase in residual strain. It can also be observed that a higher water-to-cement ratio (*w*/*c*) will cause more serious damage.

## 3. Analysis and Modeling

After the experimental standards and data have been reviewed and the damage indexes are discussed and analyzed, the statistical analysis and modeling work are conducted. Since the *DF* is calculated according to the decay of RDEM, then predictive models of RDEM with respect to the material inputs are necessary. Three models are adopted in the current study: the linear model, exponential model, and logic model implemented by an artificial neural network (ANN).

### 3.1. Adoptable Models

#### 3.1.1. Linear Model

The deterioration of compressive strength in FTCs has been modeled by Berto’s environmental damage parameter *d_env_*, where *f_c_* and *f_cd_* stand for the compressive strength of sound and damaged concrete, respectively, as shown in Equation (1) [54]. Based on ATSM C666-03 (A) [54,55], the environmental damage parameter *d_env_* could be calculated as Equation (2), where *ζ* was a parameter depending on the original compressive strength of concrete before FTCs, and *N_eq_* was named the equivalent number of FTCs. The calculation of *N_eq_* is shown in Equation (3) [54]:

(1) $$d_{env}=1-f_{cd}/f_{c}$$

(2) $$d_{env}=\zeta N_{eq}$$

(3) $$N_{eq}=\chi N^{\beta }$$

where *χ* and *β* were parameters relating to experimental conditions. Substitute Equations (2) and (3) into Equation (1) and introduce a new parameter *α* = *ζ·χ*. The prediction model of relative compressive strength is given in Equation (4), which is assumed also applicable for RDEM since two damage indexes have a certain correlation (see Figure 9). In fact, the statistical analysis (described in next Section 3.2) shows that the RDEM has a clear tendency while relative compressive strength does not. In addition, RDEM is adopted to derive *DF*, so it is valuable to apply such a prediction model in terms of RDEM. In Equation (4), *α* and *β* are parameters that depend on concrete strength (*f_c_*) and experimental conditions (*w*/*c*, *T_min_*). Relative value is used in this model to eliminate the effect of different shapes and sizes of the specimens. Once *α* and *β* are determined under certain environmental conditions and material properties, this power relationship will turn into a linear model based on the statistical analysis, which will be discussed later.

(4) $$\begin{array}{l} f_{cd}/f_{c}=1-\alpha N^{\beta }\\ RDEM=1-\alpha N^{\beta } \end{array}$$

#### 3.1.2. Exponential Model

Mutluturk et al. proposed a decay function model to describe the integrity loss of rocks when subjected to freezing–thawing cycles [56]. The degradation was modeled as a first-order process, meaning that the disintegration rate is proportional to the rock integrity before each cycle. As shown in Equation (5), *dI*/*dN* is the rate of integrity reduction, *λ* is the decay constant, and *N* is the number of cycles. Then, by integrating Equation (5) within the range of *I*_0_ and *I_N_* (the integrity at the beginning and after *N* cycles, respectively), the relation can otherwise be expressed in a logarithmic form as shown in Equation (6), and finally, an exponential form as shown in Equation (7). The term *e^-λN^* is the decay factor, which indicates the proportion of the remaining index after *N* cycles, and constant *λ* represents the relative integrity loss during each single cycle. The decay function model can be adjusted to describe the accumulation of frost damage in concrete by substituting *I_N_*/*I*_0_ with RDEM and is demonstrated in Equation (8).

(5) −dI/dN=λI

(6) $$\ln(I_{0}/I_{N})=\lambda N$$

(7) $$I_{N}/I_{0}=e^{-\lambda N}$$

(8) RDEM=exp(−λN)

Until now, two rough prediction models (linear and exponential) of RDEM degradation under the effect of frost damage have been proposed, as shown in Equations (4) and (8). The constant *α*, *β*, and *λ* in the equations, which are concerned with variables listed in Table 2, remain undetermined at this stage.

#### 3.1.3. Artificial Neural Network Model

**Figure 12:**
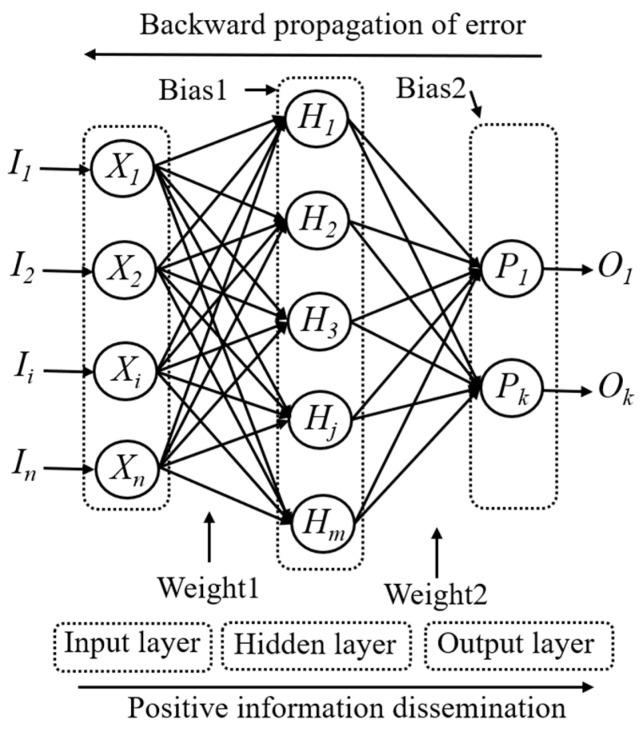
The structure of multi-layer back-propagation neural networks.

**Figure 13:**
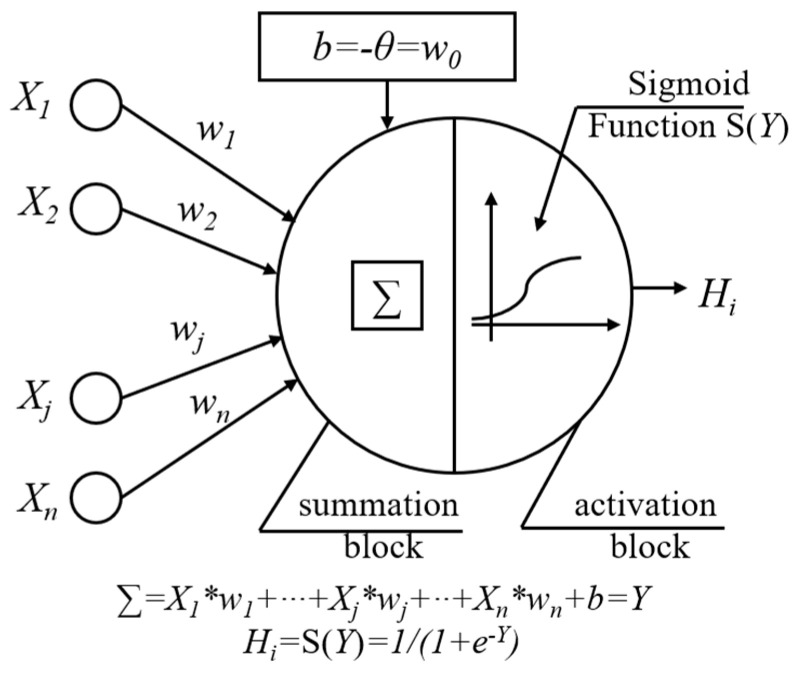
Artificial neuron model of BPNNs.

In recent years, artificial neural networks have been widely used in civil engineering and other disciplines such as composites, geotechnical, energy, biomedical, and so on [57,58,59,60,61,62]. In this paper, the artificial neural networks will be used to explore the relationship between the parameters discussed above and the properties of concrete. Artificial neural networks whose structures are layered are composed of interconnected processing elements called neurons, which can be trained to operate a given input and thus provide a desired output. There are several types of ANN algorithms, but the most widely used in civil engineering is the multi-layer back-propagation neural networks (BPNN), which is also employed in this research [63]. There are three kinds of BPNN layers, i.e., the input layer which receives signals from outside and transfers them into inside layers, the hidden layer which processes data, and the output layer which generates predicted results. Each neuron in BPNN interacts with each other via weighted connections as shown in Figure 12. The model of the artificial neuron of BPNNs is shown in Figure 13. Models are built and trained by using the Neural Network Fitting subroutine of MATLAB.

The way that BPNNs “learn” the relationship between input data and output data is through modifying the connection weights and bias to reduce the errors between them. This is carried out by minimizing the mean squared error (MSE) using the Levenberg–Marquardt backpropagation algorithm in this research. The data collected will be randomly divided into three parts, i.e., the training part which is presented to the network during training and used to adjust the network according to its error, the validation part which is used to measure network generalization and halt training when generalization stops improving, and the testing part which has no effect on training and is only used to measure network performance during and after training.

### 3.2. Regression and Discussion

#### 3.2.1. Data Selection and Pretreatment

**Figure 14:**
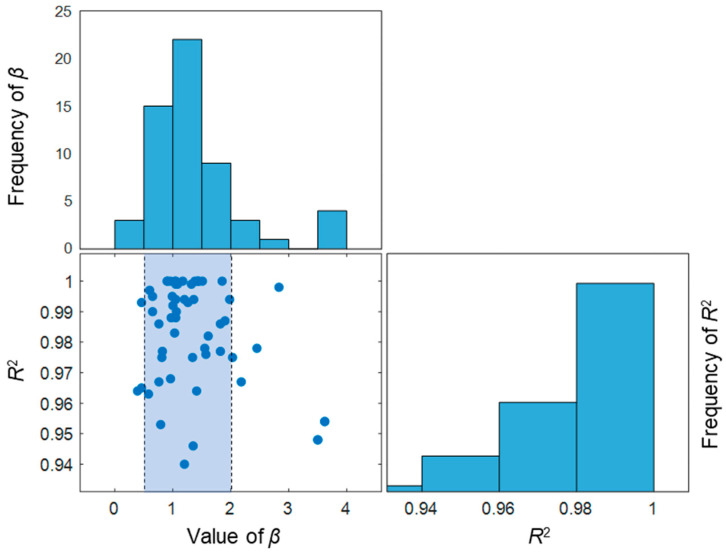
Distribution of *β* in the power function and the selected range for analysis.

In order to obtain the factors (*α*, *β*) of the linear model under different experimental conditions (*w*/*c*, original compressive strength), the collected results were organized carefully as follows: (1) by regression, the experimental results whose *β* was beyond the range of 0.5–1.5 were removed (see Figure 14); (2) set *β* as 1 for the sake of convenience, which causes the power relationship to be a linear model; (3) the value of *α* could be obtained by regression, and *R^2^* were calculated accordingly; (4) results with *R^2^* lower than 0.90 were removed. The procedure is illustrated in Figure 14: the subfigure at the upper-left corner shows the frequency of *β* whose values gather around 1. Therefore, 1 was taken as the fixed value of *β*, which caused the model to be linear. The scattered values of corresponding *R^2^* are illustrated with a bar chart at the right-bottom corner. The subfigure at the bottom left shows the values of *R^2^* against the values of *β*. The blue area signifies the values of *β* within the range of 0.5–2.0. The values of *β* outside this area will not be considered. After such process, *β* was simplified to be 1, and *α* became the only parameter left. In this linear model, *α* represents the reduction in RDEM loss in each cycle.

#### 3.2.2. Regression Result and Discussion

**Figure 15:**
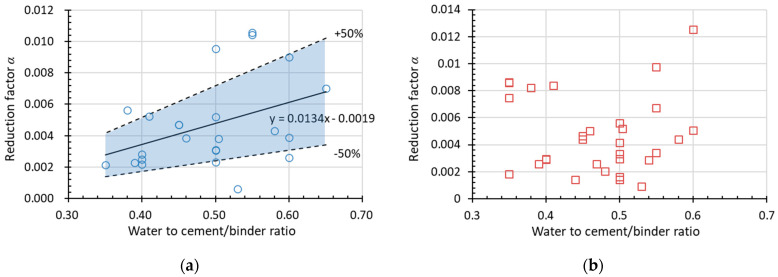
Factor *α* in the linear function in terms of *w*/*c* for (**a**) RDEM and (**b**) *Rf_c_* of non-air-entrained concrete.

**Figure 16:**
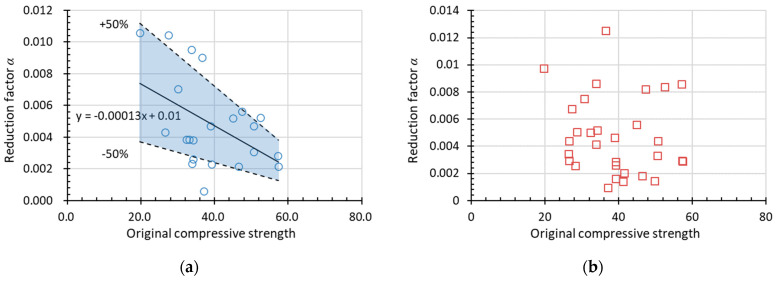
Factor *α* in the linear function in terms of original compressive strength for (**a**) RDEM and (**b**) *Rf_c_* of non-air-entrained concrete.

Figure 15 and Figure 16 show the experimental data used to obtain *α* (assuming *β* = 1) for RDEM and the relative compressive strength (*Rf_c_*) of non-air-entrained concrete in terms of the water to cement/binder ratio and the original compressive strength, respectively. The dotted lines above and below represent *α* with a deviation of ±50%. Most of the data presented here stay within the ±50% range (the blue area), with only a few data out-of-range; thus, the relationship between the reduction factor and the water-to-cement ratio is believed to regress rationally by linear formulas as indicated in the figures. Therefore, it is concluded that *α* increases with the increase in *w*/*c* and decreases with the improvement of the original compressive strength. Meanwhile, it is found that no clear tendency exists if using relative compressive strength as the damage index, as shown in Figure 15b and Figure 16b. However, the prediction of relative compressive strength can be obtained from the prediction model of RDEM and the relationship between both damage indexes, as already stated in Section 2.

**Figure 17:**
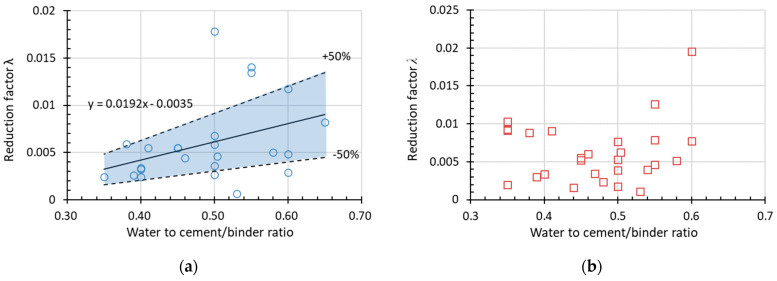
Factor *λ* in the linear function in terms of *w*/*c* for (**a**) RDEM and (**b**) *Rf_c_* of non-air-entrained concrete.

**Figure 18:**
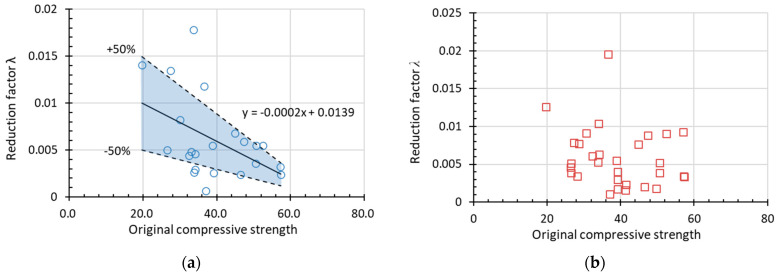
Factor *λ* in the linear function in terms of original compressive strength for (**a**) RDEM and (**b**) *Rf_c_* of non-air-entrained concrete.

In the decay (exponential) function, the value of *λ* for RDEM at different *w*/*c* and original compressive strengths can be regressed following a similar manner as for *α*. As drawn in Figure 17 and Figure 18, *λ* in this decay function indicates the speed of degradation and shows a similar trend as *α* does. By comparison, it can be concluded that parameter *λ* is more affected by *w*/*c* and original compressive strength than parameter *α* is. Additionally, *w*/*c* has a more significant influence than original compressive strength whether it is *α* or *λ* since the slopes in Figure 15 and Figure 17 are larger than that in Figure 16 and Figure 18. Again, it is found that factor *λ* for relative compressive strength does not have a clear tendency, as Figure 17b and Figure 18b.

Satisfactory agreement could be found for most of the data. Results that fall out of the ±50% range are regarded as ineffective and thus left out of consideration. This fact strongly demonstrates the applicability of the proposed models.

#### 3.2.3. Influencing Factors on Deterioration Speed

(1) Effect of *w*/*c* ratio

**Figure 19:**
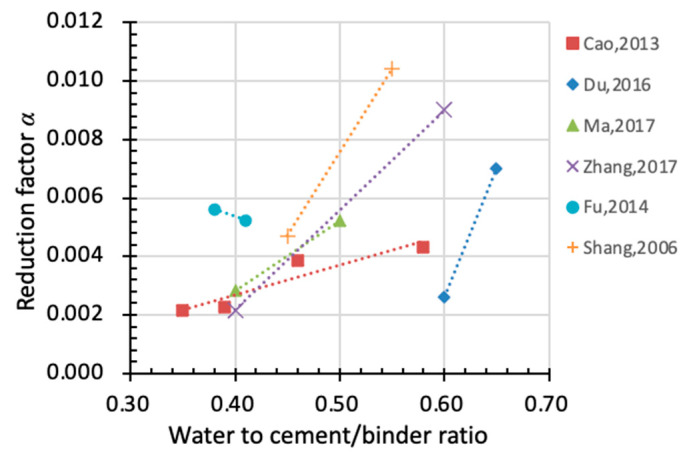
Relationship between *w*/*c* ratio and reduction factor α (x-axis: water to cement ratio, y-axis: factor α).

According to Gong et al. [64], different water-to-cement ratios may lead to considerable influence on the pore size distribution of cementitious materials, thus affecting the ice formation process during freezing–thawing cycles. A higher *w*/*c* ratio means a larger proportion of capillary pores and a bigger volume of ice formation during freezing. As shown in Figure 19, a larger *w*/*c* ratio leads to a higher value of factor *α* (except for Fu’s results), suggesting that faster damage accumulation takes place in concrete materials. The value of *α* varies greatly with *w*/*c* ratios, and even the same *w*/*c* ratio (e.g., *w*/*c* = 0.6) yields values of *α* that are poles apart in different studies. This indicates that there are other factors that influence *α* at the same time. Comparing Figure 17 and Figure 19, it further proves that the experimental data from the individual study are scattered due to the different material properties and freeze–thaw conditions by the standard. Therefore, it is reasonable to put sufficient test data to empirically model the reduction factor instead of adopting only specific data.

(2) Effect of original compressive strength

**Figure 20:**
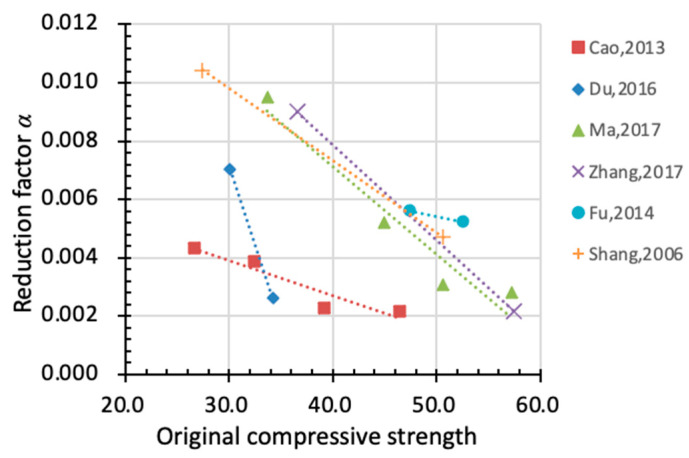
Relationship between original compressive strength and reduction factor *α* (x-axis: original compressive strength, y-axis: factor *α*).

Concrete with higher original strength is more compacted with fewer internal defects. This indicates a smaller volume of pore and less ice formation during freezing–thawing cycles. Figure 20 illustrates the effect of original compressive strength on the reduction in *α*. As the original compressive strength improves, *α* drops accordingly, suggesting a slower rate of degradation and better performance in durability.

(3) Effect of entrained air

**Figure 21:**
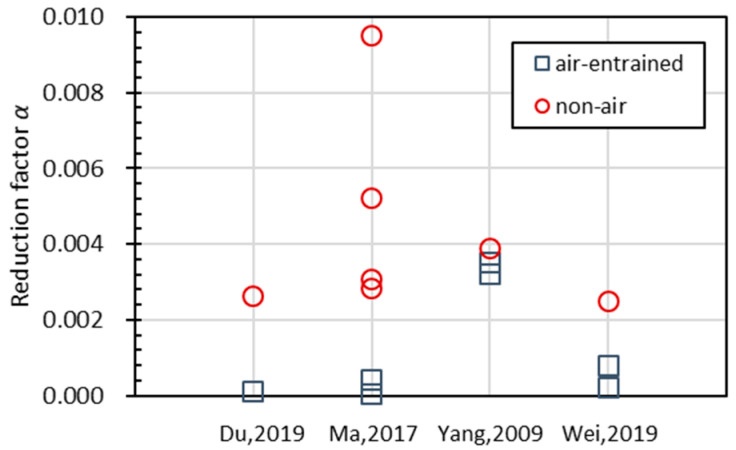
The reduction speed *α* for air-entrained and non-air-entrained concrete.

Due to the fact that different agents were used in different experiments to introduce air voids into the material, and the quality of entrained voids was difficult to assess, it is almost impossible to consider the effect of entrained air quantitatively at this stage. However, according to the data available (see Figure 21), it can be observed that the reduction factor is significantly lower for air-entrained specimens, meaning a slower deterioration speed and a higher resistance capacity. Entrained air in the material improves the resistance of concrete to freezing–thawing cycles, but the quantitative conclusion requires the careful assessment of air voids [65,66,67,68], and further statistical analysis will cover that in the future.

(4) Effect of lowest temperature

**Figure 22:**
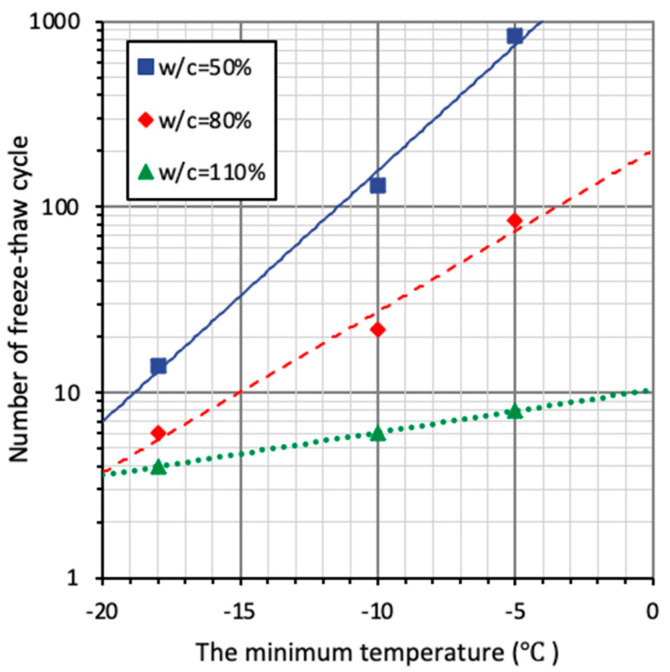
Number of FTC when RDEM drops to 60% for different lowest temperatures.

Ishii et al. [69] conducted freezing-thawing tests on concrete up to one thousand temperature cycles, with the water-to-cement ratio and minimum temperature as variables. As shown in Figure 22, the horizontal axis is the minimum temperature and the vertical axis is the number of freezing–thawing cycles when the material is damaged (i.e., the relative dynamic elastic modulus of the specimen drops below 60%). It can be observed that a higher water-to-cement ratio (*w*/*c*) and lower minimum temperature (*T_min_*) lead to a faster deterioration of speed and thus more severe frost damage. According to the experiment results presented in Figure 22, specimens with the same *w*/*c* (0.49) were damaged at the 14th and 673th cycle when the minimum temperatures were −6 °C and −18 °C, respectively. From −6 °C to −18 °C, the decrease in the minimum temperature resulted in a dramatical 48-times-faster degradation process, which indicated that the effect of the minimum temperature cannot be neglected. However, given the fact that Ishii et al.’s research was based on hydraulic structures, the *w*/*c* chosen in the experiment was higher than concrete used in ordinary buildings. Therefore, the conclusions derived here require further research.

#### 3.2.4. Analysis Based on ANN

In this research, the compressive strength, *w*/*c*, and air content of concrete were considered as the input variables, whereas *α* or *λ* was considered as the output variables. In order to obtain more suitable results and obtain a faster convergence in the training process, it is recommended to scale the databases in a range of (−1,1) [70]. The input and output variables used in the training process can be obtained by

(9) $$X_{n}=2\times \frac{X-X_{\min}}{X_{\max}-X_{\min}}-1$$

where *X* is the original data of the sample, *X_n_* is the normalized value of the sample, and *X_min_* and *X_max_* are the minimum and maximum values of the interested parameter.

After removing the isolated points of normalized variables that deviate from the others, there are 24 datasets for the BPNN model training, including 15 datasets for training (65%), 4 datasets for validation (15%), and 5 datasets for testing (20%). The data for training and testing are picked randomly from databases in every machine learning loop, which follows the logic provided by the ANN subroutine. One hidden layer for BPNNs, whose activation function is the Sigmoid Function, was used in this research, as many studies [71,72,73] showed that a neural network with one hidden layer can obtain a good result. The linear output neurons were used for the output layer.

**Figure 23:**
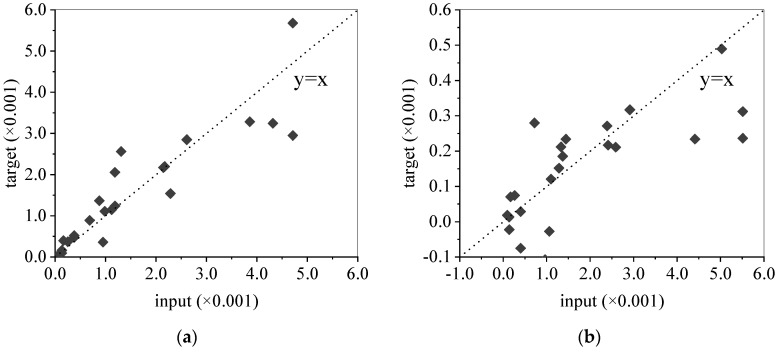
The relationship between predicted values and original values of the whole dataset: (**a**) for *α*, (**b**) for *λ*.

The training results of different parameters are shown in Table 3, where *R* values measure the correlation between outputs and targets. The *R* value of 1 means a close relationship, while 0 means a random relationship. The relationship between the predicted values and original values of the whole dataset for two parameters is shown in Figure 23. Obviously, the training effect of parameter *α* is better than that of parameter *λ*. Therefore, Equation (4) has more regularity and a better effect in characterizing the freezing–thawing damage of concrete than Equation (8).

### 3.3. Durability Factor

To assess the resistance to the freezing–thawing cycles of concrete, the durability factor (*DF*) has been recommended by ASTM C666-03 [23] to describe the relative durability of materials after different FTCs. The *DF* can be calculated as shown in Equation (10):

(10) $$DF=\frac{P_{N}N}{M}$$

where *DF* is the durability factor under freezing–thawing for the test specimen; *P_N_* (%) is the relative dynamic modulus of elasticity (RDEM) after the Nth freeze–thaw cycle; *N* is the number of cycles at which the RDEM reaches the specified minimum value (usually 60%); *M* is the maximum number of cycles at which the exposure is to be terminated (usually 300 cycles).

However, it is unrealistic to directly take up *DF* as a general indicator for materials tested in distinctive experimental conditions different from the method in ASTM C666-03, especially for those in service in real structures. In fact, the formula of the *DF* actually describes a simple linear behavior of the frost deterioration but just using two states (starting and ending points) to describe it. In order to extend the utilization of the *DF*, it is correlated to the reduction factor *α* in the linear model based on the collected date, as shown in Equation (11):

(11) $$DF=\left\{\begin{array}{ll} 1-300\alpha & \;\alpha \leq 1.33\times 10^{-3}\;\\ \frac{1}{1250\alpha }\; & \;\alpha >1.33\times 10^{-3}\; \end{array}\right.$$

**Figure 24:**
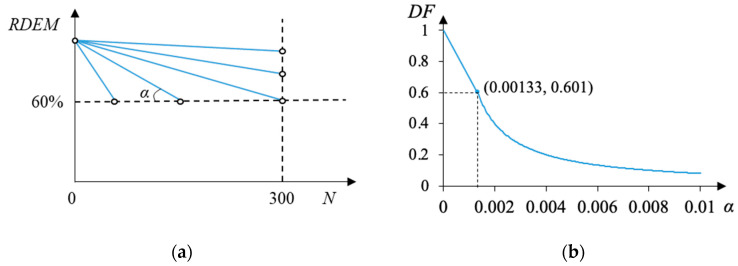
The mathematic relation between the durability factor (*DF*) and linear deterioration speed–*α*, (**a**) Definition of *DF* and corresponding *α*; (**b**) continuous relation between *DF* and *α*.

**Figure 25:**
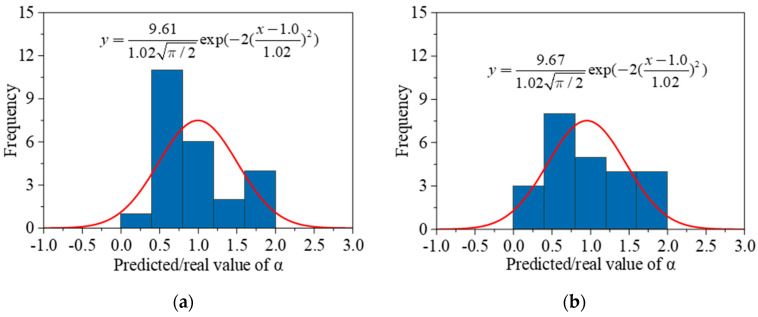
The probability distribution of prediction accuracy in terms of *α*, (**a**) model prediction from *w*/*c*; (**b**) model prediction from original compressive strength.

It is observed that the *DF* decreases linearly with the increase in *α* at first, and then the reduction rate turns slower after *α* reaches 1.33 × 10^−3^. Factor *α* reflects the average RDEM loss during each freezing–thawing cycle, as illustrated in Figure 24. Since factor *α* is closely related to different experimental parameters (e.g., water/cement ratio, air ratio, and compressive strength) and the predictive models have been proposed in Figure 15a and Figure 16a, the established relationship between *α* and the *DF* provides a potential alternative to evaluate the deterioration rate of different concrete materials. In other words, once *α* is determined according to material inputs, the DF can be conveniently calculated to roughly estimate the durability of the concrete material, and a comparison between concrete under different conditions can be easily drawn. Besides, the *DF* shall be related to not only the material properties but also environmental factors such as the freezing–thawing conditions including minimum temperature. However, the minimum temperatures in all the available experimental data are limited in a small range as shown in Table 2. Therefore, it should be emphasized that the *DF* prediction model proposed by the current study ignores the environmental impact by only considering the material inputs. The probability distribution of the prediction accuracy is plotted in Figure 25, where the horizontal axis means the ratio between the predicted decay factor and the experimentally obtained value, and the vertical axis shows the corresponding number of experimental data.

### 3.4. Further Discussions

Regarding the application and limitation of the models proposed in this study, it shall be noted that the reduction factors are derived based on the range of 0.35–0.65 for the water-to-cement ratio and 20–60 MPa for the compressive strength in order to satisfy the deviation. This range is also where the most experimental data are located. However, in real applications, the *w*/*c* of *f_c_* may be out of scope, which may require further modification or careful attention to apply the models.

**Figure 26:**
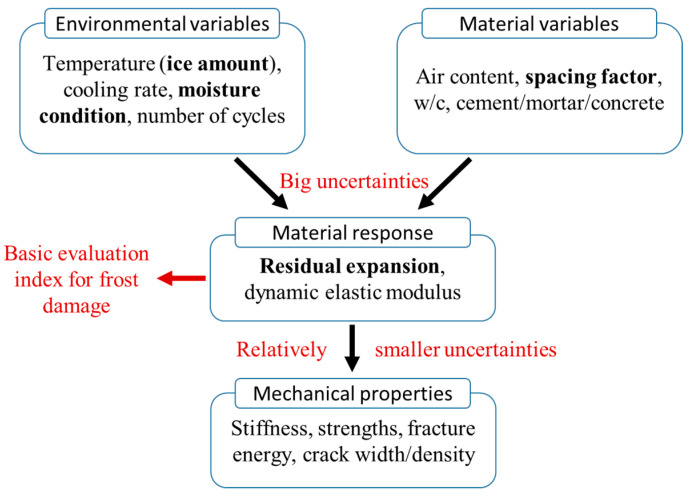
The influential factors, logic scheme, and uncertainties in the frost damage prediction.

Moreover, as shown in Figure 26, uncertainties caused by environmental and material variables lead to a big scattering of experimental results and raise considerable difficulty in the establishment of a universally applicable prediction model. Among these influencing factors, the most sensible ones are the ice amount, moisture conditions, and void spacing factor. Although, in most experimental studies, the tested specimens are supposed to be fully saturated, and then air bubbles are not considered if an AE agent is not used. However, based on the previous theoretical works [10,14], the internal pore pressure during FTC is very sensitive to the saturation degree and air distribution. For example, a slight change in the water content at a highly saturated state from 95% to 100% can cause a big difference in the freezing pressures, and the actual water content is difficult to control depending on the curing conditions and pre-saturation treatments before FTC starts. Further, the entrapped air during the mixing process, or the included air by a water-reducing agent, may also affect the actual spacing factor [74], thus introducing big uncertainties in the frost resistivity. That is why among the collected data in this study, even the material mixture and experimental setup are similar, different researchers may obtain quite scattering results.

Therefore, based on the attempts of the study, if aiming to predict frost damage progress with time, it seems that the scattering of FTC experiments is even more dominant than the logic rules of the frost mechanism. Thus, it may be more reliable to adopt theoretical and numerical models to obtain the general predicting formulae; see the authors’ previous work [29].

Finally, if ignoring the frost history, and only focusing on the material properties after frost damage, relatively smaller uncertainties exist between the frost-damage indexes and macro mechanical properties (Figure 26). At the mesoscale of material, the frost damage embodies itself as the degradation of basic evaluation indexes like residual expansion and dynamic elastic modulus; thus, the residual expansion and RDEM can be the basic indexes to evaluate the frost damage level, and the further macro mechanical properties (stiffness, strength, fracture energy, crack width/density) can be linked to these basic indexes with rather smaller uncertainties [17,28,30].

Therefore, for the practical evaluation of frost-damaged behaviors, the overall evaluation can be achieved after measuring one or two indexes (residual strain, RDEM, or compressive strength), while for the time-dependent frost damage progress, more logical ways are needed, which will be investigated in the future.

## 4. Conclusions

In this paper, the numbers of freezing–thawing experimental data were collected, reviewed, and analyzed comparatively. The consistency of the deterioration pattern can be found in testing data of the same source, while variation in environmental conditions and material parameters in different studies means it is difficult to establish a reliable and quantified index to describe the damage appropriately. Therefore, it is recommended to conduct the statistical analysis with numerous data based on which empirical model is proposed rather than adopting only specific experimental results. The main conclusions are as follows:

(1) Experimental conditions and material parameters have significant effects on the behavior of concrete subjected to freezing–thawing tests that are conducted under the guidance of different testing standards. The relationship between damaged indexes and the number of cycles shows a reasonable tendency, where a larger water-to-cement ratio and lower original strength cause faster deterioration with more FT cycles. Yet, a lack of consistency between results from different works is substantial. Correlations among damaged properties were carefully analyzed and stable results can be obtained.
(2) Two prediction models of RDEM degradation under the effect of frost damage were proposed, namely, the linear model and the exponential model. Briefly, ±50% accuracy can be achieved after proper selection and pretreatment with data. This further proves the scattering nature of the experimental data and the importance of applying the statistical analysis. Moreover, a model based on an Artificial Neural Network was also suggested in this paper; although it can give nice predicting results, the authors do not recommend this way due to a lack of physical and logical meaning.
(3) To quantify the frost resistance ability of different materials, the durability factor (*DF*) was discussed in the paper. The correlation between the *DF* and the reduction factor *α* serves as an alternative index to evaluate the resistivity of materials. The *DF* can be regarded as a material characteristic which has the physical meaning of frost resistance of the concrete. This will help to evaluate the experiments that do not follow the standard testing requirements. Besides, this factor of the *DF* can be easily used and understood by the engineering application to judge the frost resistance, even though the standard test is not exactly followed. Further research will be needed to figure out the response of basic evaluation indexes under various experimental conditions.
(4) For the practical evaluation of frost-damaged behaviors, the overall evaluation can be achieved after measuring one or two indexes (residual strain, RDEM, or compressive strength), while for the time-dependent frost damage progress, it seems that the scattering of FTC experiments is even more dominant than the logic rules of the frost mechanism. Thus, it may be more reliable to adopt theoretical and numerical models to obtain the general predicting formulae, which will be conducted in the future.

## Figures and Tables

**Table 1:** Freezing-thawing conditions and tested values in various standards.

Standard	Procedure	Period per Cycle	Temperature Cycle [°C]	Preparation of Specimens	Size of Specimens	Measurements after Certain Cycles	Assessment of Damage [%] ^1^	Damage Criterion
ASTM C666-03	A: freezing and thawing in water; B: freezing in air thawing in water	2~5 h	−18~+4 °C	Molded beam: curing for 14 days Beam specimens sawed from hardened concrete: immersing in saturated lime water at 23.0 ± 2.0 °C for 48 h	≥75 mm, ≤125 mm in width, depth, or diameter; ≥275 mm, ≤405 mm in length.	(1) fundamental transverse frequency; (2) mass loss; (3) length change	*DF = PN*/*M*	(1) 300 cycles; (2) RDEM below 60%; (3) length expansion larger than 0.1%
JIS A1148:2010	A: freezing and thawing in water; B: freezing in air thawing in water	3~4 h	−18~+5 °C	Curing for 28 days	100 × 100 × 400	(1) fundamental transverse frequency; (2) mass loss;	*DF = PN*/*M*	(1) 300 cycles; (2) RDEM below 60%;
RILEM TC 176-IDC	freezing and thawing in air	12 h	−20~+20 °C	Curing for 14 days→surface drying for 21 days→pre-saturation for 7 days	150 × 110 × 70	(1) scaling mass loss; (2) moisture uptake; (3) ultrasonic transit time	Value of *N* when *R_u,n_* reaches 80%, where *R_u,n_* = 𝜏*_n_*^2^ and 𝜏*_n_*^2^ = (*t_cs_*−*t_c_*)/(*t_n_*−*t_c_*)	*R_u,n_* reaching 80%
GB/T 50082–2009 (GBJ82-85)	freezing and thawing in water	2~4 h	−18~+5 °C	Curing for 24 days→pre-saturation for 4 days	100 × 100 × 400	(1) fundamental transverse frequency; (2) mass loss;	*F*: Value of *N* when RDEM reaches 60% or mass loss 5%	(1) Expected cycles; (2) RDEM below 60%; (3) mass loss larger than 0.1%

^1^*DF*: durability factor of the test specimen, *P*: relative dynamic modulus of elasticity at *N* cycles, %, *N*: number of cycles at which *P* reaches the specified minimum value for discontinuing the test or the specified number of cycles at which the exposure is to be terminated, whichever is less, *M*: specified number of cycles at which the exposure is to be terminated, *R_u,n_*: relative change of dynamic modulus of elasticity of ultrasonic transit time after *N* freezing–thawing cycles, *t_cs_*: the total transit time at the end of capillary suction (*cs*), in ms before the first freeze–thaw cycle, *t_n_* is the total transit time after *N* freezing–thawing cycles (FTCs), in ms, *t_c_*: is the transit time in the coupling medium, in ms, *F*: frost resistance factor.

**Table 2:** Collected freezing-thawing data in laboratory tests.

	Standard	Size [mm^3^]	FTC Exposure [°C] ^2^	No. of FTCs	*w*/*c* (*w*/*b*)	f_c_ [MPa]	Air Void [%] ^3^	Measured Properties ^1^
Shi [33] (1997)	ASTM C666-84	Φ100 × 203	10~−30	0, 30, 60, 90	0.35	61.71	/ (AE)	*f_c_*, *E*, *v*, *f_ts_*, *τ_s_*, *G*
						59.85		
					0.48	43.37		
						38.63		
Hasan [17] (2004)	ASTM C666-03	100 × 100 × 200	20~−25	0, 88, 148, 188	0.60	47.8	4.5 (AE)	*ε_pf_*, *f_c_*, *E*, *ε_c_*_0_
				0, 50, 100, 200, 300	0.50	49.8	1.5 ^3^	
Shang [20] (2006)	GBJ82-85	100 × 100 × 100	6~−15	0, 25, 50, 75	0.50	34.2	1.7	RDEM, Δ*W*, *f_ts_*, *f_c_*, *E*, *ε_c_*_0_
Ji [34] (2007)	GBJ82-85	100 × 100 × 100	8~−17	0, 15, 30, 50	0.50 (0.35)	33.98	1.9	*f_c_*, *E*, *ε_c_*_0_, *f_ts_*
		150 × 150 × 150				30.71		
Cao [35] (2013)	GBJ82-85	150 × 150 × 150	8~−17	0, 25, 50, 75,100, 125	0.41 (0.35)	50.7	3.0	RDEM, *f_c_*, *E*, *ε_c0_*, *a*, *b*
					0.44 (0.39)	45.4	3.4	
					0.55 (0.46)	35.6	2.6	
					0.75 (0.58)	29.1	3.0	
Diao [36] (2011)	GBJ82-85	100 × 100 × 300	20~−35	0, 50, 100, 150, 200	0.44	41.30	/ (Non-AE)	*f_c_*, *E*
					0.50	26.59		
					0.55	26.48		
Xu [37] (2019)	-	100 × 100 × 100 400 × 100 × 100	6~−16	0, 25, 50, 75, 100, 125, 150, 175, 200, 225, 250, 275, 300	0.44	42.18	/ (AE)	RDEM, *f_c_*, Δ*W*, *ε_c_*_0_
					0.40	51.57		
					0.35	62.92		
Fu [38] (2014)	GB/T 50082-2009	100 × 100 × 100	5~−17	0, 5, 10, 15, 20	0.38	47.39/14.55	/ (AE/Non-AE)	RDEM, *f_c_*
					0.35	57.12/25.33		
					0.41	52.51/16.5		
Li [39] (2019)	-	100 × 100 × 100 400 × 100 × 100	5~−18	0, 50, 100, 150, 200	0.45	38.6	0.7	RDEM, *f_c_*, Δ*W*
						37.2	1.3	
						35.8	2.4	
						33.1	3.1	
						30.3	4.7	
						26.8	6.3	
						21.2	7.4	
Yang [40] (2009)	-	300 × 100 × 100	8~−16	0, 25, 50, 75, 100, 125	0.6	33.15	/ (AE/ Non-AE)	RDEM, *f_c_*, *f_ts_*, *f_f_*
						31.08		
						30.65		
Xiao [41] (2010)	GBJ82-85	100 × 100 × 100 400 × 100 × 100	5~−18	0, 50, 100, 150, 200, 250, 300	0.35	61.85	3.7	RDEM, *f_c_*, Δ*W*
					0.45	49.74	4.4	
					0.55	32.09	4.5	
Wei [42] (2003)	GBJ82-85	100 × 100 × 100	20~−15	0, 50, 100, 150, 200, 250, 300	0.40	-	/ (AE/ Non-AE)	RDEM, *f_c_*
					0.32			
					0.50 (0.40)			
					0.43 (0.34)			
Shang [43] (2006)	GBJ82-85	100 × 100 × 100 150 × 150 × 150 400 × 100 × 100	5~−18	0, 50, 100, 150, 200, 250, 300, 350 400	0.45	50.65/38.9	0.9/1.9	RDEM, *f_c_*, *E*, *f_ts_*, *f_f_*, *f_t_*, Δ*W*, *ε_c_*_0_
					0.55	27.41/19.66	1.9	
					0.40	34.2/26.3	5.5~6.5	
Zandi [44] (2009)	RILEM TC 176-IDC	Φ100 × 100 Φ100 × 200 Φ100 × 250	20~−20	-	0.57	40.58	/ (Non-AE)	*f_c_*, *E*, *f_ts_*
Shang [45] (2009)	GBJ82-85	100 × 100 × 100 400 × 100 × 100	8~−17	0, 100, 200, 300, 400	0.40	26.3	/ (AE)	RDEM, *f_c_*, *E*, *f_ts_*, *ΔW*
Zhang [46] (2017)	GB/T 50082–2009	100 × 100 × 100 400 × 100 × 100	8~−15	0, 10, 50, 100	0.40	57.41	1.4	RDEM, *f_c_*
					0.60	36.58/33.50	2.1/5.2	
Ma [47] (2017)	ASTM C666-97	100 × 100 × 100 400 × 100 × 100	8~−17	0, 50, 100	0.40	57.2/43.56	/ (AE/ Non-AE)	RDEM, *f_c_*, *G_f_*
					0.50	44.92/37.41		
					0.50	33.69/50.60		
Hasan [48] (2002)	ASTM C666-97	400 × 100 × 100	4.4~−17.8	0, 10, 17,28, 34, 41, 62, 76, 109, 205, 261, 305	0.5	-	/ (AE/Non-AE)	RDEM, *f_t_*, *w_max_*, *G_f_* , *E_t_*
Du [49] (2019)	GB/T 50082–2009	100 × 100 × 100 400 × 100 × 100	5~−18	0, 50, 100, 150, 200, 250, 300	0.6	34.2	2.8	RDEM, *ε_r_*, Δ*W*
						27.4	4.5	
						22.8	6.9	
Fan [50] (2015)	GBJ82-85	400 × 100 × 100	4~−18	0, 25, 50, 100, 125	0.5	33.89	/ (Non-AE)	RDEM, *f_c_*, Δ*W*
Guan [51] (2019)	GB/T 50082–2009	100 × 100 × 100 300 × 100 × 100	20~−18	0, 50, 100, 150, 200, 250, 300	0.53	37.1	/ (Non-AE)	RDEM, *f_c_*, Δ*W*, *ε_c_*_0_
Hao [52] (2018)	GB/T 50082–2009	100 × 100 × 100 400 × 100 × 100	8~−15	0, 25, 50, 75, 100	0.50	39.3	/ (Non-AE)	RDEM, *f_c_*, Δ*W*, *f_ts_*
Duan [29] (2011)	GB/T 50082–2009	100 × 100 × 300	8~−17	0, 75, 100, 125, 150	0.60	28.73	/ (Non-AE)	RDEM, *f_c_*, *ε_r_*, *ε_c_*_0_
				0, 100, 175, 200	0.54	39.18		
				0, 50, 75, 100, 125, 150	0.48	41.54		
Du [53] (2016)	GB/T 50082–2009	400 × 100 × 100	8~−19	0, 25, 50, 75, 100 125, 150	0.50	39.3	2.5	RDEM, *E, ε_r_*, Δ*W*
					0.60	34.2	2.8	
						27.4	4.5	
					0.65	30.1	3.2	

^1^ *f_c_*: compressive strength, *E*: Elastic modulus, *E_t_*: secant elastic modulus, *ε_c_*_0_: compressive peak strain, *ε_r_*: residual strain, *f_ts_*: splitting tensile strength, *f_t_*: pure tensile strength, *ff*: flexural strength, *τ_s_*: shear strength, *G*: shear modulus, RDEM: relative dynamic elastic modulus, Δ*W*: weight loss, *w_max_*: maximum crack width, *G_f_*: fractural energy, *a* and *b*: parameters in Guo et al.’s compressive stress–strain model [47]; ^2^ The FTC exposure is “water condition” in default unless “in air” is specifically marked; ^3^ The air void shown in only numbers represents the “Non-AE” condition.

**Table 3:** Performance of the BPNN model for different parameters.

Parameter	*N* ^1^	MSE			R			
		Train	Val	Test	Train	Val	Test	All
*α*	11	0.0649	0.0506	0.1167	0.9140	0.9143	0.9277	0.9053
*λ*	11	0.1457	0.2172	0.3251	0.8048	0.8574	0.8288	0.7396

^1^ *N*: the number of neurons of the hidden layer in the BPNN model.

## Data Availability

Data is contained within the article.

## Appendix A

**Table A1:** Selected data of RDEM during FTC and the regression in terms of cycles (N).

ID	*w*/*c*	*f_c_*	Air Void [%]	*T*_min_	*y* = 1 − *αN**^β^*					*y* = exp(−*λN*)		*DF* ^1^	*DF* ^2^	Ref.
					α	β	R^2^	α (β = 1)	R^2^	λ	R^2^			
1	0.50	34.2	(non-AE)	−15	8.9 × 10^−3^	0.81	0.97	3.8 × 10^−3^	0.96	4.6 × 10^−3^	0.97	0.20	0.21	Shang, 2006 [36]
2	0.75	26.6	3.6 (non-AE)	−17	4.4 × 10^−3^	1.00	0.99	4.3 × 10^−3^	0.99	5.0 × 10^−3^	0.99	-	0.19	Cao, 2013 [38]
3	0.55	32.4	3.4 (non-AE)	−17	3.4 × 10^−3^	1.03	0.98	3.9 × 10^−3^	0.98	4.4 × 10^−3^	0.98	-	0.21	Cao, 2013 [38]
4	0.44	39.2	2.6 (non-AE)	−17	1.7 × 10^−3^	1.06	0.99	2.3 × 10^−3^	0.99	2.6 × 10^−3^	0.98	-	0.35	Cao, 2013 [38]
5	0.41	46.5	3 (non-AE)	−17	1.7 × 10^−3^	1.05	0.99	2.1 × 10^−3^	0.99	2.4 × 10^−3^	0.99	-	0.37	Cao, 2013 [38]
6	0.60	34.2	2.8 (non-AE)	−20	4.3 × 10^−4^	1.41	0.96	2.6 × 10^−3^	0.94	2.9 × 10^−3^	0.92	-	0.31	Du, 2019 [52]
7	0.60	27.4	4.5 (AE)	−20	3.7 × 10^−7^	***2.18***	0.97	2.5 × 10^−4^	***0.84***	2.6 × 10^−4^	***0.83***	0.90	0.92	Du, 2019 [52]
8	0.60	22.8	6.9 (AE)	−20	1.3 × 10^−5^	1.35	0.95	8.9 × 10^−5^	0.92	9.0 × 10^−5^	0.92	0.97	0.97	Du, 2019 [52]
9	0.50	33.9	(non-AE)	−20	9.0 × 10^−4^	1.20	0.94	2.3 × 10^−3^	0.93	2.6 × 10^−3^	0.92	-	0.35	Fan, 2015 [53]
10	0.53	37.1	(non-AE)	−18	7.6 × 10^−4^	0.96	0.97	6.1 × 10^−4^	0.97	6.6 × 10^−4^	0.97	0.80	0.82	Guan, 2019 [54]
11	0.50	39.3	(non-AE)	−18	1.6 × 10^−2^	***0.46***	0.99	1.6 × 10^−3^	***0.82***	1.7 × 10^−3^	***0.85***	-	0.50	Hao, 2018 [55]
12	0.50	-	(non-AE)	−17.8	1.0 × 10^−2^	0.79	0.95	3.1 × 10^−3^	0.93	5.8 × 10^−3^	0.97	0.14	0.26	Hasan, 2002 [51]
13	0.40	57.2	(non-AE)	−17	3.7 × 10^−4^	1.45	1.00	2.8 × 10^−3^	0.97	3.2 × 10^−3^	0.95	-	0.28	Ma, 2017 [50]
14	0.50	44.9	(non-AE)	−17	9.4 × 10^−4^	1.38	1.00	5.2 × 10^−3^	0.98	6.8 × 10^−3^	0.94	0.16	0.15	Ma, 2017 [50]
15	0.40	43.6	(AE)	−17	8.2 × 10^−6^	1.17	1.00	1.8 × 10^−5^	1.00	1.8 × 10^−5^	1.00	-	0.99	Ma, 2017 [50]
16	0.50	37.4	(AE)	−17	6.6 × 10^−4^	0.90	1.00	4.2 × 10^−4^	1.00	4.2 × 10^−4^	1.00	-	0.88	Ma, 2017 [50]
17	0.50	33.7	(non-AE)	−17	1.4 × 10^−2^	0.91	1.00	9.5 × 10^−3^	1.00	1.8 × 10^−2^	0.96	0.08	0.08	Ma, 2017 [50]
18	0.50	50.6	(non-AE)	−17	2.5 × 10^−3^	1.05	1.00	3.1 × 10^−3^	1.00	3.6 × 10^−3^	0.99	-	0.26	Ma, 2017 [50]
19	0.40	57.4	1.4 (non-AE)	−15	2.2 × 10^−4^	1.51	1.00	2.2 × 10^−3^	0.97	2.4 × 10^−3^	0.95	-	0.37	Zhang, 2017 [49]
20	0.60	36.6	2.1 (non-AE)	−15	1.1 × 10^−2^	0.96	1.00	9.0 × 10^−3^	1.00	1.2 × 10^−2^	1.00	0.09	0.09	Zhang, 2017 [49]
21	0.60	33.5	5.2 (AE)	−15	7.3 × 10^−3^	***0.39***	0.96	4.9 × 10^−4^	***0.69***	5.1 × 10^−4^	***0.70***	-	0.85	Zhang, 2017 [49]
22	0.40	34.2	(AE)	−15	1.8 × 10^−10^	***3.50***	0.95	4.1 × 10^−4^	***0.71***	4.3 × 10^−4^	***0.70***	0.94	0.88	Shang, 2009 [48]
23	0.40	26.3	(AE)	−15	1.8 × 10^−10^	***3.50***	0.95	4.1 × 10^−4^	***0.71***	4.3 × 10^−4^	***0.70***	0.94	0.88	Shang, 2009 [48]
24	0.45	50.7	0.9 (non-AE)	−18	8.0 × 10^−4^	1.43	1.00	4.7 × 10^−3^	0.97	5.5 × 10^−3^	0.94	0.15	0.17	Shang, 2006 [46]
25	0.55	27.4	1.9 (non-AE)	−18	7.7 × 10^−3^	1.08	1.00	1.0 × 10^−2^	1.00	1.3 × 10^−2^	0.97	0.08	0.08	Shang, 2006 [46]
26	0.45	38.9	0.9 (non-AE)	−18	8.0 × 10^−4^	1.43	1.00	4.7 × 10^−3^	0.97	5.5 × 10^−3^	0.94	0.15	0.17	Shang, 2006 [46]
27	0.55	19.7	1.9 (non-AE)	−18	9.2 × 10^−3^	1.04	1.00	1.1 × 10^−2^	1.00	1.4 × 10^−2^	0.98	0.08	0.08	Shang, 2006 [46]
28	0.40	34.2	5.5–6.5 (AE)	−18	8.4 × 10^−11^	***3.62***	0.95	3.8 × 10^−4^	***0.73***	4.0 × 10^−4^	***0.72***	0.94	0.89	Shang, 2006 [46]
29	0.40	26.3	5.5–6.5 (AE)	−18	8.4 × 10^−11^	***3.62***	0.95	3.8 × 10^−4^	***0.73***	4.0 × 10^−4^	***0.72***	0.94	0.89	Shang, 2006 [46]
30	0.40	-	(non-AE)	−15~−20	2.6 × 10^−3^	0.99	0.99	2.5 × 10^−3^	0.99	3.4 × 10^−3^	0.97	0.3	0.32	Wei, 2003 [45]
31	0.32	-	(non-AE)	−15~−20	4.2 × 10−8	***2.83***	1.00	1.1 × 10^−3^	***0.80***	1.2 × 10^−3^	***0.76***	0.58	0.68	Wei, 2003 [45]
32	0.50	-	(AE)	−15~−20	9.4 × 10^−4^	0.97	0.99	7.9 × 10^−4^	0.99	8.8 × 10^−4^	0.99	0.76	0.76	Wei, 2003 [45]
33	0.43	-	(AE)	−15~−20	7.4 × 10^−4^	0.76	0.99	2.0 × 10^−4^	0.96	2.1 × 10^−4^	0.96	0.94	0.94	Wei, 2003 [45]
34	0.35	61.8	3.7 (AE)	−18	3.5 × 10^−4^	0.82	0.98	1.3 × 10^−4^	0.96	1.3 × 10^−4^	0.96	0.96	0.96	Xiao, 2010 [44]
35	0.45	49.7	4.4 (AE)	−18	2.5 × 10^−3^	***0.46***	0.97	1.3 × 10^−4^	***0.76***	1.4 × 10^−4^	***0.76***	0.96	0.96	Xiao, 2010 [44]
36	0.55	32.1	4.5 (AE)	−18	1.5 × 10^−3^	0.60	1.00	1.6 × 10^−4^	0.89	1.7 × 10^−4^	0.90	0.95	0.95	Xiao, 2010 [44]
37	0.60	33.1	(non-AE)	−16	3.0 × 10^−3^	1.05	0.99	3.9 × 10^−3^	0.99	4.8 × 10^−3^	0.97	0.21	0.21	Yang, 2009 [43]
38	0.60	31.1	(AE)	−16	1.4 × 10^−3^	1.20	0.99	3.5 × 10^−3^	0.98	4.2 × 10^−3^	0.96	0.22	0.23	Yang, 2009 [43]
39	0.60	30.6	(AE)	−16	6.2 × 10^−4^	1.36	0.99	3.2 × 10^−3^	0.97	3.8 × 10^−3^	0.94	0.24	0.25	Yang, 2009 [43]
40	0.45	38.6	0.7 (non-AE)	−18	7.5 × 10^−7^	***2.45***	0.98	1.3 × 10^−3^	***0.82***	1.4 × 10^−3^	***0.78***	-	0.61	Li, 2019 [42]
41	0.45	37.2	1.3 (AE)	−18	9.8 × 10^−6^	***1.90***	0.99	1.2 × 10^−3^	***0.88***	1.3 × 10^−3^	***0.84***	-	0.64	Li, 2019 [42]
42	0.45	35.8	2.4 (AE)	−18	5.8 × 10^−5^	1.55	0.98	1.2 × 10^−3^	0.92	1.4 × 10^−3^	0.89	0.62	0.64	Li, 2019 [42]
43	0.45	33.1	3.1 (AE)	−18	5.0 × 10^−5^	1.57	0.98	1.1 × 10^−3^	0.91	1.3 × 10^−3^	0.88	0.63	0.66	Li, 2019 [42]
44	0.45	30.3	4.7 (AE)	−18	1.1 × 10^−5^	***1.82***	0.98	9.8 × 10^−4^	***0.87***	1.1 × 10^−3^	***0.84***	0.67	0.71	Li, 2019 [42]
45	0.45	26.8	6.3 (AE)	−18	1.1 × 10^−5^	***1.82***	0.99	9.5 × 10^−4^	***0.88***	1.1 × 10^−3^	***0.85***	0.68	0.71	Li, 2019 [42]
46	0.45	21.2	7.4 (AE)	−18	4.1 × 10^−6^	***1.98***	0.99	8.7 × 10^−4^	***0.87***	9.6 × 10^−4^	***0.84***	0.67	0.74	Li, 2019 [42]
47	0.38	47.4	(non-AE)	−17	2.2 × 10^−3^	1.34	0.97	5.6 × 10^−3^	0.95	5.9 × 10^−3^	0.95	-	0.14	Fu, 2014 [41]
48	0.35	57.1	(non-AE)	−17	3.7 × 10^−4^	***2.03***	0.98	6.8 × 10^−3^	***0.87***	7.1 × 10^−3^	***0.85***	-	0.12	Fu, 2014 [41]
49	0.41	52.5	(non-AE)	−17	9.5 × 10^−4^	1.61	0.98	5.2 × 10^−3^	0.93	5.5 × 10^−3^	0.92	-	0.15	Fu, 2014 [41]
50	0.38	14.6	(AE)	−17	1.7 × 10^−2^	0.76	0.97	8.9 × 10^−3^	0.94	9.7 × 10^−3^	0.95	-	0.09	Fu, 2014 [41]
51	0.35	25.3	(AE)	−17	1.6 × 10^−2^	0.65	0.99	6.1 × 10^−3^	0.93	6.5 × 10^−3^	0.94	-	0.13	Fu, 2014 [41]
52	0.41	16.5	(AE)	−17	2.2 × 10^−2^	0.65	0.99	8.4 × 10^−3^	0.94	9.1 × 10^−3^	0.95	-	0.10	Fu, 2014 [41]
53	0.44	42.2	(AE)	−16	3.1 × 10^−4^	1.05	1.00	4.1 × 10^−4^	1.00	4.3 × 10^−4^	1.00	0.87	0.88	Xu, 2019 [40]
54	0.40	51.6	(AE)	−16	5.2 × 10^−5^	1.26	0.99	2.1 × 10^−4^	0.97	2.2 × 10^−4^	0.97	0.93	0.94	Xu, 2019 [40]
55	0.35	62.9	(AE)	−16	2.5 × 10^−5^	1.32	1.00	1.4 × 10^−4^	0.97	1.4 × 10^−4^	0.97	0.95	0.96	Xu, 2019 [40]
56	0.50	40.7	2.5 (non-AE)	−18	4.9 × 10^−3^	0.58	0.96	6.8 × 10^−4^	0.85	7.2 × 10^−4^	0.87	-	0.79	Du, 2016 [57]
57	0.65	30.1	3.2 (non-AE)	−18	2.8 × 10^−4^	1.85	1.00	7.0 × 10^−3^	0.93	8.2 × 10^−3^	0.89	-	0.11	Du, 2016 [57]

^1^ 23 groups (out of 57) cannot apply the standard *DF*; ^2^ *DF*: Based on the equation *DF* = 1 − 300*α*, when *α* ≤ 1.33 × 10^−3^; DF = 0.0008/*α*, when *α* ≥ 1.33 × 10^−3^; Values in bold and italic means eliminated groups with poor tendency.
